# Albumin–Butyrylcholinesterase as a Novel Prognostic Biomarker for Hepatocellular Carcinoma Post-hepatectomy: A Retrospective Cohort Study with the Hiroshima Surgical Study Group of Clinical Oncology

**DOI:** 10.1245/s10434-024-16650-6

**Published:** 2024-12-10

**Authors:** Takeshi Tadokoro, Tsuyoshi Kobayashi, Naruhiko Honmyo, Shintaro Kuroda, Masahiro Ohira, Masakazu Hashimoto, Koichi Oishi, Akihiko Oshita, Tomoyuki Abe, Takashi Onoe, Toshihiko Kohashi, Hideki Ohdan

**Affiliations:** 1 Department of Gastroenterological and Transplant Surgery, Graduate School of Biomedical and Health Science, Hiroshima University, Hiroshima, Japan; 2 HiSCO: Hiroshima Surgical Study Group of Clinical Oncology, Hiroshima, Japan; 3 Department of Gastroenterological Surgery, Hiroshima Prefectural Hospital, Hiroshima, Japan; 4 Department of Surgery, Chugoku Rosai Hospital, Kure, Japan; 5 Department of Surgery, Onomichi General Hospital, Onomichi, Japan; 6 Department of Surgery, National Hospital Organization Higashihiroshima Medical Center, Higashihiroshima, Japan; 7 Department of Surgery, National Hospital Organization Kure Medical Center and Chugoku Cancer Center, Kure, Japan; 8 Department of Surgery, Hiroshima City North Medical Center, Asa Citizens Hospital, Hiroshima, Japan

**Keywords:** Albumin, Butyrylcholinesterase, Hepatocellular carcinoma, ABC, Discovery cohort, Validation cohort

## Abstract

**Background:**

This study aimed to investigate the association between a new biomarker that incorporates albumin (Alb) and butyrylcholinesterase (BCHE) levels, as well as the prognosis of hepatocellular carcinoma (HCC) after hepatectomy.

**Methods:**

The study enrolled 1712 patients who underwent primary hepatectomy for HCC between January 2003 and December 2019 at seven institutions belonging to the Hiroshima Surgical Study Group of Clinical Oncology. The entire dataset was randomly split into discovery and validation cohorts in a 7:3 ratio. The product of the preoperative Alb and BCHE levels was defined as the ABC. In the discovery cohort, the patients in the high-ABC group (≥ 951) were compared with those in the low-ABC group (< 951). These findings then were confirmed in the validation cohort.

**Results:**

In the discovery cohort, a significant difference was observed in the 5-year survival rate between the high- and low-ABC groups (*p* < 0.001), and ABC was identified as an independent prognostic factor for HCC. Similarly, in the validation cohort, a significant difference was observed in the 5-year survival rate between the high- and low-ABC groups (*p* < 0.001), and ABC was identified as an independent prognostic factor for HCC. Furthermore, in the discovery and validation cohorts, significant differences in the early recurrence rate between the two groups were observed (*p* < 0.001 and *p* = 0.020, respectively).

**Conclusions:**

For patients with HCC, ABC is a useful predictive biomarker because it can be calculated in a simple manner and because it provides accurate prognostic information.

**Supplementary Information:**

The online version contains supplementary material available at 10.1245/s10434-024-16650-6.

Hepatocellular carcinoma (HCC) is the sixth most common malignancy and the fourth most common cause of cancer-related death worldwide.^1^ Although liver resection is a safe and potentially curative treatment for HCC, the recurrence rate of HCC after curative hepatic resection is high, and long-term survival rates remain unsatisfactory.^2^ Despite advances in surgical techniques and perioperative management, many patients experience tumor recurrence, leading to poor overall prognosis and limited survival benefits. Therefore, better risk assessment of HCC prognosis and recurrence is important for making informed treatment decisions.

Nutritional status, inflammation, and cancer are closely associated with each other. Sarcopenia, malnutrition, and cancer cachexia are highly prevalent and can occur concurrently or separately, particularly in older patients with cancer.^3^ Nutrition-related diseases and disorders cause a decline in tumor immunity, leading to postoperative complications and cancer progression. Furthermore, cancer-related systemic inflammation promotes catabolism in patients, contributing to metastasis.^4^

Several biomarkers of nutritional status such as the geriatric nutritional risk index (GNRI), the prognostic nutritional index (PNI), and the controlling nutritional status (CONUT) score can predict long-term outcomes in various cancers.^5–7^ These indices are also useful for evaluating the prognosis of HCC.^8–10^ The Child–Pugh and albumin–bilirubin (ALBI) scores, which reflect liver function, are also known to be predictive of long-term prognosis for patients with HCC.^11,12^ However, some of these indices involve complex calculations and cannot be considered simple scoring systems.

Albumin (Alb), a protein, and butyrylcholinesterase (BCHE), an enzyme, are synthesized in the liver and reflect its functional reserve. In particular, recent studies have shown that decreased levels of BCHE are associated with poor prognosis in several cancers (e.g., pancreatic, gastric, and colorectal cancers).^13–15^ Furthermore, findings have shown the combination of serum Alb and BCHE levels to be useful for predicting the prognosis for patients with colorectal cancer because this biomarker is related to preoperative nutritional status.^16^

In this study, we hypothesized that the combination of serum Alb and BCHE, reflecting both nutritional status and liver function, could serve as a simple indicator for the prognosis of patients with HCC. Therefore, we performed this multi-institutional retrospective analysis aiming to investigate the prognostic efficacy of a combination of preoperative serum Alb and BCHE levels, which we designed as a new biomarker of the prognosis for patients who underwent hepatic resection for HCC.

## Methods

### Study Design

This study enrolled 1712 patients who underwent primary hepatectomy for HCC between January 2003 and December 2019 at seven institutions belonging to the Hiroshima Surgical Study Group of Clinical Oncology (HiSCO). The study was conducted in accordance with the principles of the Declaration of Helsinki. The study protocol was approved by the Ethics Committee of our hospital (E2015-0026) based on the Ethical Guidelines for Clinical Research of the Ministry of Health, Labor and Welfare in Japan. The requirement for written informed consent was waived, and informed consent was obtained from all the patients or members of their families by an opt-out approach at each institution due to the retrospective nature of the study.

### Data Collection

After enrollment, the entire study population was randomly divided into discovery (70%) and validation (30%) cohorts, after which surgical margin-positive cases were excluded in each cohort. The number of cases with surgical margin positivity was 61 in the discovery cohort and 37 in the validation cohort. The discovery cohort was used to develop a model to predict postoperative prognosis, whereas the validation cohort was used to validate the model.

The investigated patient characteristics included age, sex, body mass index (BMI), and hepatitis virus status. The following blood test parameters were examined: hemoglobin, platelet count, albumin, BCHE, aspartate aminotransferase (AST), alanine aminotransferase (ALT), total bilirubin, prothrombin activity (PT), indocyanine green retention rate at 15 min (ICGR15), and glycated hemoglobin (HbA1c). The examined tumor and surgical factors included alpha fetoprotein (AFP), des-γ-carboxy prothrombin (DCP), tumor number, tumor size, tumor differentiation, microvascular invasion, external hepatic metastasis, surgical procedure, operative time, and blood loss.

### Definition of ABC

The ABC was defined as the product of preoperative Alb and BCHE levels (Alb × BCHE). The ABC cutoff value was set to 951 according to receiver operating characteristic (ROC) analysis of overall survival (OS) at the 5-year follow-up evaluation in the discovery cohort. The patients with an ABC lower than 951 (low-ABC group, *n* = 584); were compared with those who had an ABC of 951 or higher (high-ABC group,* n* = 553) to assess the rates of OS and recurrence-free survival (RFS). The same analyses were performed in the validation cohort.

### Nutritional and Liver Function Assessments

Nutritional status and liver function, were assessed using five general indices: GNRI, PNI, CONUT score, Child–Pugh classification, and ALBI grade. The ALBI score formula was as follows:

$${\text{ALBI}}\;{\text{score}} = 0.{669} \times {\text{log}}_{{{1}0}} \left( {{\text{total}}\;{\text{bilirubin}}\left[ {\upmu {\text{mol}}/{\text{L}}} \right]} \right) - 0.0{85} \times \left( {{\text{Alb}}\left[ {{\text{g}}/{\text{L}}} \right]} \right).$$

The ALBI score was graded as follows: grade 1 (− 2.60 or less), grade 2 (− 2.59 to − 1.39), and grade 3 (greater than − 1.39).^17^ The PNI was calculated as follows:^18^

$${\text{PNI}} = {1}0 \times {\text{Alb}}\left( {{\text{g}}/{\text{dL}}} \right) + 0.00{5} \times {\text{lymphocyte}}\;{\text{count}}\left( {/{\text{mm}}^{{3}} } \right).$$

The GNRI was calculated using the following equation:^19^

$${\text{GNRI}} = {14}.{89} \times {\text{Alb}}\left( {{\text{g}}/{\text{dL}}} \right) + {41}.{7} \times \left( {{\text{body}}\;{\text{weight}}/{\text{ideal}}\;{\text{body}}\;{\text{weight}}} \right).$$

The ideal body weight was defined as height (m)^2^ × 22. Preoperative CONUT scores were calculated based on serum Alb concentration, peripheral lymphocyte count, and total cholesterol concentration. The Alb concentrations ≥ 3.5, 3.0–3.49, 2.5–2.99, and < 2.5 g/dL were scored as 0, 2, 4, and 6 points, respectively. The total lymphocyte counts ≥ 1600, 1200–1599, 800–1199, and < 800/mm^3^ were scored as 0, 1, 2, and 3 points, respectively, and the total cholesterol concentrations ≥ 180, 140–179, 100–139, and < 100 mg/dL were scored as 0, 1, 2, and 3 points, respectively. The CONUT score was defined as the sum of these components.^20^

### Statistical Analyses

Continuous variables are presented as medians (interquartile ranges), and nominal variables are expressed as numbers (%). The Student’s *t* test and the Mann–Whitney *U* test were performed for continuous variables, whereas the chi–square test and Fisher’s exact test were performed for nominal variables to compare the two groups. The prognostic capabilities of ABC, GNRI, PNI, CONUT score, Child–Pugh score, and ALBI grade were compared using ROC curves and area under the curve (AUC) for OS and RFS at the 5-year follow-up evaluation in the discovery cohort. The cutoff values for these indices were determined using ROC curve analysis of OS at the 5-year follow-up evaluation in the discovery cohort. The Kaplan–Meier method was used to analyze OS and RFS, and the survival rate was calculated using the log-rank test. Multivariate analyses were performed to assess factors influencing OS and RFS using the Cox regression model for factors with a *p* value lower than 0.05 in univariate analysis.

Statistical significance was set at a *p* value lower than 0.05. All analyses were performed using JMP Pro 17 software (SAS Institute, Cary, NC, USA).

## Results

### Discovery Cohort

Table 1 shows the clinicopathologic characteristics of the patients in the low- and high-ABC groups in the discovery cohort. Age, number of men, and BMI were significantly lower (all *p* < 0.001) in the low-ABC group than in the high-ABC group. Additionally, the numbers of patients with hepatitis B or C were significantly higher in the low-ABC group than in the high-ABC group (*p* = 0.002).

**Table 1:** Comparison of the clinicopathologic characteristics between the low- and high-ABC groups in the discovery cohort

Variable		Low-ABC group (*n* = 584)	High-ABC group (*n* = 553)	*p* Value
Median age	Years (IQR)	68 (62–75)	71 (64–76)	< 0.001
Sex, male	*n* (%)	409 (70.3)	447 (80.8)	< 0.001
Median BMI	kg/m^2^ (IQR)	22.6 (20.6–24.7)	23.6 (21.5–26.0)	< 0.001
Viral hepatitis status	*n* (%)	418 (71.9)	348 (63.2)	0.002
Median hemoglobin	g/dL (IQR)	12.6 (11.4–13.8)	14.1 (13.1–15.2)	0.183
Median platelet count	× 10^4^/μL (IQR)	12.1 (8.8–18.1)	16.0 (12.2–20.2)	< 0.001
Median albumin	g/dL (IQR)	3.7 (3.4–3.9)	4.3 (4.1–4.5)	< 0.001
Median butyrylcholinesterase	U/L (IQR)	184 (150–213)	286 (257–320)	< 0.001
Median AST	IU/L (IQR)	40 (28–56)	29 (23–41)	< 0.001
Median ALT	IU/L (IQR)	32 (21–50)	28 (18–42)	< 0.001
Median total bilirubin	mg/dL (IQR)	0.8 (0.6–1.0)	0.8 (0.6–1.0)	0.083
Median prothrombin activity	% (IQR)	84 (76–93)	92 (84–101)	< 0.001
Median ICGR15	% (IQR)	17.7 (11.4–26.5)	11.2 (8.0–16.5)	< 0.001
Median HbA1c	% (IQR)	5.7 (5.1–6.5)	5.8 (5.4–6.5)	0.197
Child–Pugh classification, B	*n* (%)	79 (13.6)	10 (1.8)	< 0.001
Median alpha fetoprotein	ng/mL (IQR)	12.7 (5.0–75.8)	7.0 (4.1–45.0)	0.378
Median DCP	mAU/mL (IQR)	47 (21–386)	49 (23–321)	0.019
Median ALBI score (IQR)		− 2.42 (− 2.62 to − 2.14)	− 2.91 (− 3.10 to − 2.70)	< 0.001
Median GNRI (IQR)		96.8 (92.3–99.8)	105.7 (102.7–108.7)	< 0.001
Median PNI (IQR)		43.9 (40.1–47.2)	51.2 (48.1–53.9)	< 0.001
Median CONUT score (IQR)		1 (0–2)	3 (2–4)	< 0.001
Multiple tumors	*n* (%)	173 (30.2)	139 (25.4)	0.072
Median tumor size	mm (IQR)	25 (18–40)	25 (18–40)	0.295
Poorly differentiated or undifferentiated	*n* (%)	65 (11.5)	53 (10.0)	0.429
Microvascular invasion	*n* (%)	58 (10.4)	48 (9.1)	0.445
Extrahepatic metastasis	*n* (%)	5 (0.9)	5 (0.9)	0.942
Liver cirrhosis	*n* (%)	181 (37.1)	91 (20.0)	< 0.001
Open liver resection	*n* (%)	518 (88.7)	475 (85.9)	0.155
Surgical procedure				
Partial resection	*n* (%)	278 (47.6)	193 (35.0)	< 0.001
Segmentectomy or sectionectomy	*n* (%)	245 (42.0)	310 (56.2)	< 0.001
Hemi-hepatectomy or tri-sectionectomy	*n* (%)	83 (14.2)	74 (13.4)	0.694
Median operative time	Min (IQR)	271 (210–342)	297 (231–371)	0.001
Median blood loss	mL (IQR)	250 (122–522)	244 (100–466)	0.027

ABC, Albumin × butyrylcholinesterase; BMI, Body mass index; AST, Aspartate aminotransferase; ALT, Alanine aminotransferase; ICGR15, Indocyanine green retention at 15 min; HbA1c, Glycated hemoglobin; DCP, Des-γ-carboxy prothrombin; ALBI, Albumin–bilirubin; GNRI, Geriatric nutritional risk index; PNI, Prognostic nutrition index; CONUT, Controlling nutritional risk status

The high-ABC group showed significantly higher platelet counts, Alb levels, BCHE levels, and PT than the low-ABC group (all *p* < 0.001). The low-ABC group showed significantly higher AST, ALT, and ICGR15 as well as a higher proportion of Child–Pugh class B classifications than the high-ABC group (all *p* < 0.001). The ALBI score was significantly higher in the low-ABC group than in the high-ABC group (*p* < 0.001), whereas GNRI, PNI, and CONUT score were significantly higher in the high-ABC group than in the low-ABC group (all *p* < 0.001).

The tumor marker DCP was significantly lower in the low-ABC group than in the high-ABC group (*p* = 0.019). The number of patients with a diagnosis of liver cirrhosis on non-neoplastic liver tissue in resected specimens was significantly higher in the low-ABC group than in the high-ABC group (*p* < 0.001). The high-ABC group had a significantly longer operative time (*p* = 0.001) but a significantly lower blood loss (*p* = 0.027) than the low-ABC group.

Regarding the surgical procedure, the number of patients who underwent partial resection was significantly higher in the low-ABC group, whereas the number of patients who underwent segmentectomy or sectionectomy was significantly higher in the high-ABC group (*p* < 0.001 for all). The groups did not differ in terms of hemoglobin levels, total bilirubin levels, HbA1c, AFP, multiple tumors, tumor size, tumor differentiation, microvascular invasion, extrahepatic metastasis, open liver resection, or the number of patients who underwent hemi-hepatectomy or tri-sectionectomy.

Figure S1 presents Alb and BCHE, and Fig. S2 shows a comparison of the nutritional and liver functional indices for the OS. The ROC analysis showed that the AUC for ABC was 0.685, which was higher than those for Alb (0.677) and BCHE (0.673). Additionally, the AUCs for other indices were as follows: Child–Pugh score (0.601), ALBI score (0.675), PNI (0.661), GNRI (0.677), and CONUT score (0.636). The AUC for the ABC was the highest. The ROC curves for RFS were constructed using the same indices, and the AUCs for ABC (0.670), Child–Pugh score (0.588), ALBI score (0.662), PNI (0.651), GNRI (0.663), and CONUT score (0.627) were evaluated. The AUC of ABC was the highest for RFS as well (Fig. S3).

**Fig. 1:**
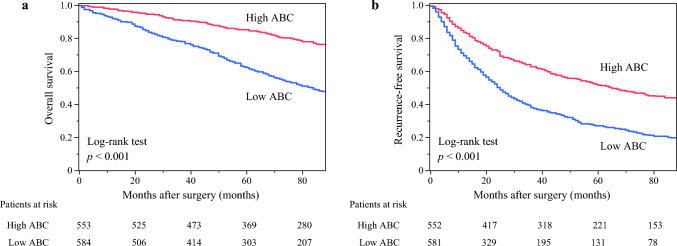
Kaplan–Meier curves of (**a**) overall and (**b**) recurrence-free survival in the discovery cohort. ABC, albumin × butyrylcholinesterase

Figure 1 shows the results of the Kaplan–Meier analysis for OS and RFS using ABC. For both 5-year OS (Fig. 1a) and RFS (Fig. 1b), significant differences in rates between the low- and high-ABC groups (*p* < 0.001, respectively) were observed.

Table 2 summarizes the results of the uni- and multivariate analyses for OS and RFS. One of the covariates, major hepatectomy, is defined as the removal of three or more Couinaud segments.

**Table 2:** Prognostic factors of overall and recurrence-free survival in the discovery cohort

Variables		Overall survival						Recurrence-free survival					
		Univariate analysis			Multivariate analysis			Univariate analysis			Multivariate analysis		
		HR	95% CI	*p* Value	HR	95% CI	*p* Value	HR	95% CI	*p* Value	HR	95% CI	*p* Value
Age > 0	Years	1.61	1.34–1.93	< 0.001	1.77	1.29–2.43	< 0.001	1.14	0.99–1.31	0.072			
Sex, male		0.86	0.71–1.06	0.161				1.10	0.93–1.30	0.255			
BMI > 25	kg/m^2^	0.89	0.73–1.10	0.278				0.96	0.83–1.13	0.644			
Viral hepatitis status		0.86	0.70–1.04	0.119				1.17	1.01–1.36	0.043			
ICGR15 > 15	%	1.52	1.27–1.82	< 0.001	0.98	0.71–1.35	0.883	1.51	1.31–1.73	< 0.001	1.14	0.90–1.44	0.280
Alpha fetoprotein > 10	ng/mL	1.52	1.27–1.83	< 0.001	1.30	0.95–1.78	0.097	1.53	1.33–1.76	< 0.001	1.40	1.13–1.74	0.002
DCP > 400	mAU/mL	1.56	1.27–1.91	< 0.001	1.70	1.12–2.57	0.013	1.27	1.08–1.49	0.004	1.47	1.12–1.93	0.006
Child–Pugh classification, B		2.35	1.78–3.10	< 0.001	1.62	0.99–2.64	0.054	1.72	1.36–2.19	< 0.001	1.40	0.94–2.10	0.100
Multiple tumors		1.57	1.30–1.90	< 0.001	1.33	0.97–1.82	0.076	1.97	1.70–2.29	< 0.001	1.97	1.56–2.48	< 0.001
Tumor size > 40	mm	1.33	1.09–1.63	0.006	0.83	0.51–1.35	0.453	1.12	0.95–1.32	0.175			
Poorly differentiated or undifferentiated		1.14	0.85–1.52	0.391				1.02	0.81–1.29	0.878			
Microvascular invasion		1.83	1.39–2.40	< 0.001	1.23	0.74–2.05	0.417	1.39	1.11–1.76	0.005	0.74	0.49–1.09	0.129
Extrahepatic metastasis		3.44	1.71–6.94	< 0.001	6.80	1.76–26.22	0.005	3.21	1.72–6.01	< 0.001	4.41	1.31–14.79	0.016
Liver cirrhosis		1.28	1.03–1.58	0.025	0.90	0.62–1.30	0.569	1.34	1.14–1.59	< 0.001	1.00	0.78–1.29	0.984
Open liver resection		2.20	1.46–3.32	< 0.001	1.28	0.79–2.10	0.319	1.39	1.10–1.77	0.006	1.10	0.81–1.48	0.553
Major liver resection		1.39	1.09–1.77	0.008	1.21	0.77–1.89	0.408	1.02	0.83–1.25	0.831			
Operative time > 400	Min	1.27	0.99–1.62	0.059				1.26	1.04–1.53	0.019	1.14	0.85–1.53	0.380
Blood loss > 500	mL	1.52	1.24–1.85	< 0.001	1.45	1.05–2.00	0.023	1.40	1.20–1.64	< 0.001	1.20	0.93–1.54	0.161
ABC < 951		2.55	2.10–3.09	< 0.001	1.87	1.22–2.89	0.004	1.99	1.73–2.30	< 0.001	1.43	1.07–1.91	0.016
ALBI grade ≥ 2		2.22	1.85–2.66	< 0.001	0.88	0.51–1.51	0.636	1.91	1.66–2.20	< 0.001	1.11	0.76–1.64	0.586
GNRI < 100		2.49	2.07–3.00	< 0.001	1.27	0.73–2.22	0.399	1.89	1.65–2.18	< 0.001	1.18	0.80–1.74	0.416
PNI < 45		2.34	1.79–3.07	< 0.001	1.20	0.72–1.98	0.489	1.78	1.47–2.16	< 0.001	0.99	0.69–1.42	0.955
CONUT score ≥ 3		2.19	1.66–2.89	< 0.001	1.35	0.92–1.97	0.124	1.72	1.42–2.09	< 0.001	1.22	0.93–1.61	0.154

HR, Hazard ratio; CI, Confidence interval; BMI, Body mass index; ICGR15, Indocyanine green retention rate at 15 min; DCP, Des-γ-carboxy prothrombin; ABC, Albumin × butyrylcholinesterase; ALBI, Albumin–bilirubin; GNRI, Geriatric nutritional risk index; PNI, Prognostic nutrition index; CONUT, Controlling nutritional risk status

In the univariate analysis, the significant prognostic factors for poor OS were age > 70 years, ICGR15 > 15%, AFP > 10 (ng/mL), DCP > 400 mAU/mL, Child–Pugh B classification, multiple tumors, tumor > 40 mm, microvascular invasion, extrahepatic metastasis, liver cirrhosis, open liver resection, major hepatectomy, blood loss > 500 mL, ABC < 951, ALBI grade ≥ 2, GNRI < 100, PNI < 45, and CONUT score ≥ 3 were.

In the multivariate analysis using these factors, the following five were identified as prognostic factors for poor OS: age > 70 years (hazard ratio [HR], 1.77; 95% CI 1.29–2.43; *p* < 0.001), DCP > 400 mAU/mL (HR 1.70; 95% CI 1.12–2.57; *p* = 0.013), extrahepatic metastasis (HR 6.80; 95% CI 1.76–26.22; *p* = 0.005), blood loss > 500 mL (HR 1.45; 95% CI 1.05–2.00; *p* = 0.023), and ABC < 951 (HR 1.87; 95% CI 1.22–2.89; *p* = 0.004).

In the univariate analysis, the significant prognostic factors for poor RFS included viral hepatitis status, ICGR15 > 15%, AFP > 10 ng/mL, DCP > 400 mAU/mL, Child–Pugh B classification, multiple tumors, microvascular invasion, extrahepatic metastasis, liver cirrhosis, open liver resection, operative time > 400 min, blood loss > 500 mL, ABC < 951, ALBI grade ≥ 2, GNRI < 100, PNI < 45, and CONUT score ≥ 3.

The multivariate analysis identified the factors contributing to poor RFS as follows: AFP > 10 ng/mL (HR 1.40; 95% CI 1.13–1.74; *p* = 0.002), DCP > 400 mAU/mL (HR 1.47; 95% CI 1.12–1.93; *p* = 0.006), multiple tumors (HR 1.97; 95% CI 1.56–2.48; *p* < 0.001), extrahepatic metastasis (HR 4.41; 95% CI 1.31–14.79; *p* = 0.016), and ABC < 951 (HR 1.43; 95% CI 1.07–1.91; *p* = 0.016).

### Validation Cohort

Table 3 shows a comparison of the background factors between the discovery and validation cohorts. No significant differences were observed in any of the factors between the two cohorts.

**Table 3:** Comparison of the clinicopathologic characteristics between the discovery and validation cohorts

Variable		Discovery cohort (*n* = 1137)	Validation cohort (*n* = 477)	*p* Value
Median age	Years (IQR)	70 (63–76)	70 (63–76)	0.637
Sex, male	*n* (%)	856 (75.3)	356 (74.6)	0.782
Median BMI	kg/m^2^ (IQR)	23.0 (21.0–25.3)	22.7 (20.8–25.2)	0.488
Viral hepatitis status	*n* (%)	766 (67.7)	332 (69.9)	0.381
Median hemoglobin	g/dL (IQR)	13.4 (12.1–14.6)	13.4 (12.0–14.5)	0.855
Median platelet count	× 10^4^/μL (IQR)	14.3 (10.2–19.3)	1403 (10.4–18.4)	0.724
Median albumin	g/dL (IQR)	4.0 (3.7–4.3)	4.0 (3.6–4.3)	0.912
Median butyrylcholinesterase	U/L (IQR)	233 (182–284)	236 (185–286)	0.569
Median AST	IU/L (IQR)	34 (25–49)	36 (26–51)	0.102
Median ALT	IU/L (IQR)	30 (19–46)	31 (21–48)	0.401
Median 5otal bilirubin	mg/dL (IQR)	0.8 (0.6–1.0)	0.7 (0.6–1.0)	0.196
Median prothrombin activity	% (IQR)	88 (79–97)	90 (81–98)	0.153
Median ICGR15	% (IQR)	14.0 (9.2–21.5)	13.5 (8.7–21.6)	0.659
Median HbA1c	% (IQR)	5.8 (5.3–6.5)	5.9 (5.4–6.5)	0.415
Child–Pugh classification, B	*n* (%)	89 (7.8)	30 (6.3)	0.277
Median alpha fetoprotein	ng/mL (IQR)	9.1 (5.0–57.8)	11.1 (5.0–85.7)	0.955
Median DCP	mAU/mL (IQR)	48 (22–351)	47 (22–338)	0.134
Median ALBI score (IQR)		–2.67 (–2.93 to –2.36)	–2.65 (–2.95 to –2.37)	0.672
Median GNRI (IQR)		101.3 (96.8–105.7)	101.3 (95.3–105.7)	0.912
Median PNI (IQR)		48.1 (43.7–51.9)	47.9 (44.1–52.2)	0.786
Median CONUT score (IQR)		2 (1–3)	2 (1–3)	0.563
Multiple tumors	*n* (%)	312 (27.8)	136 (29.1)	0.603
Median tumor size	mm	25 (18–40)	25 (17–40)	0.495
Poorly differentiated or undifferentiated	*n* (%)	118 (10.8)	49 (10.7)	0.929
Microvascular invasion	*n* (%)	106 (9.8)	40 (8.8)	0.553
Extrahepatic metastasis	*n* (%)	10 (0.9)	2 (0.4)	0.328
Liver cirrhosis	*n* (%)	272 (28.9)	111 (27.9)	0.715
Open liver resection	*n* (%)	993 (87.3)	426 (89.3)	0.267
Surgical procedure				
Partial resection	*n* (%)	471 (41.5)	194 (40.7)	0.769
Segmentectomy or sectionectomy	*n* (%)	555 (48.9)	232 (48.6)	0.936
Hemi-hepatectomy or tri-sectionectomy	*n* (%)	157 (13.8)	58 (12.2)	0.370
Median operative time	Min (IQR)	285 (220–357)	279 (225–355)	0.692
Median blood loss	mL (IQR)	250 (112–500)	250 (120–517)	0.801

BMI, Body mass index; AST, Aspartate aminotransferase; ALT, Alanine aminotransferase; ICGR15, Indocyanine green retention at 15 min; DCP, Des-γ-carboxy prothrombin; ALBI, Albumin–bilirubin; GNRI, Geriatric nutritional risk index; PNI, Prognostic nutrition index; CONUT, Controlling nutritional risk status

**Fig. 2:**
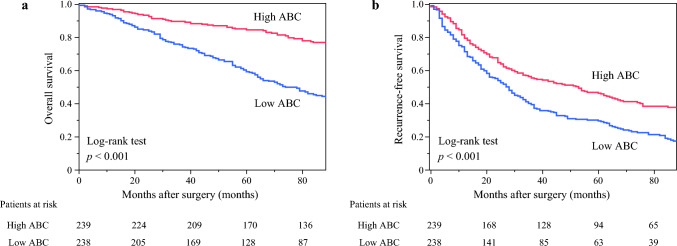
Kaplan–Meier curves of the (**a**) overall and (**b**) recurrence-free survival in the validation cohort. ABC, albumin × butyrylcholinesterase

Figure 2 shows the results from the Kaplan–Meier analysis of OS and RFS using the ABC. In both the 5-year OS (Fig. 2a) and RFS (Fig. 2b) rates, significant differences between the low- and high-ABC groups (*p* < 0.001, respectively) were observed.

Table 4 summarizes the results of the uni- and multivariate analyses for OS and RFS. In the univariate analysis, the statistically significant prognostic factors for poor OS were age > 70 years, ICGR15 > 15%, AFP > 10 ng/mL, DCP > 400 mAU/mL, Child–Pugh B classification, multiple tumors, tumor > 40 mm, microvascular invasion, open liver resection, major liver resection, ABC < 951, ALBI grade ≥ 2, GNRI < 100, PNI < 45, and CONUT score ≥ 3. In the multivariate analysis using these factors, the following three were identified as prognostic factors for poor OS: multiple tumors (HR 3.28; 95% CI 2.11–5.10; *p* < 0.001), microvascular invasion (HR 2.04; 95% CI 1.03–4.03; *p* = 0.042), and ABC < 951 (HR 1.99; 95% CI 1.02–3.89; *p* = 0.044).

**Table 4:** Prognostic factors of overall and recurrence-free survival in the validation cohort

Variables		Overall survival						Recurrence-free survival					
		Univariate analysis			Multivariate analysis			Univariate analysis			Multivariate analysis		
		HR	95% CI	*p* Value	HR	95% CI	*p* Value	HR	95% CI	*p* Value	HR	95% CI	*p* Value
Age > 70	Years	1.31	1.00–1.71	0.047	1.11	0.70–1.74	0.662	1.04	0.84–1.28	0.731			
Sex, male		1.21	0.88–1.13	0.232				1.35	1.05–1.72	0.017	1.36	1.04–1.78	0.026
BMI > 25	kg/m^2^	0.73	0.53–1.01	0.055				0.89	0.70–1.13	0.352			
Viral hepatitis status		0.96	0.72–1.28	0.772				1.00	0.80–1.26	0.971			
ICGR15 > 15	%	1.57	1.21–2.05	< 0.001	1.17	0.74–1.86	0.494	1.29	1.05–1.59	0.016	1.14	0.90–1.45	0.278
Alpha fetoprotein > 10	ng/mL	1.46	1.12–1.91	0.005	1.37	0.87–2.14	0.174	1.38	1.12–1.71	0.002	1.24	0.98–1.57	0.069
DCP > 400	mAU/mL	1.97	1.48–2.62	< 0.001	1.74	0.97–3.10	0.062	1.70	1.34–2.16	< 0.001	1.53	1.18–1.99	0.001
Child–Pugh classification, B		2.32	1.51–3.59	< 0.001	1.10	0.48–2.56	0.817	1.45	0.97–2.16	0.068	0.97	0.63–1.49	0.884
Multiple tumors		2.01	1.53–2.63	< 0.001	3.28	2.11–5.10	< 0.001	1.74	1.39–2.17	< 0.001	1.65	1.31–2.09	< 0.001
Tumor size > 40	mm	1.70	1.26–2.28	< 0.001	1.43	0.79–2.60	0.237	1.26	0.98–1.62	0.069			
Poorly differentiated or undifferentiated		1.32	0.88–1.97	0.182				1.55	1.12–2.15	0.008	1.52	1.07–2.16	0.019
Microvascular invasion		1.65	1.07–2.55	0.023	2.04	1.03–4.03	0.042	1.39	0.96–2.00	0.080			
Extrahepatic metastasis		3.17	0.78–12.79	0.106				24.47	5.78–103.67	< 0.001	30.31	6.95–132.21	< 0.001
Liver cirrhosis		1.36	0.99–1.87	0.057				1.23	0.96–1.58	0.096			
Open liver resection		2.59	1.28–5.26	0.008	1.14	0.48–2.71	0.772	1.71	1.15–2.56	0.009	1.43	0.92–2.22	0.114
Major liver resection		1.50	1.04–2.16	0.03	1.29	0.62–2.68	0.502	1.27	0.94–1.73	0.122			
Operative time > 400	min	1.08	0.74–1.56	0.700				1.06	0.80–1.43	0.672			
Blood loss > 500	mL	1.19	0.89–1.61	0.243				1.23	0.97–1.55	0.088			
ABC < 951		2.48	1.88–3.28	< 0.001	1.99	1.02–3.89	0.044	1.59	1.30–1.97	< 0.001	1.44	1.04–1.99	0.027
ALBI grade ≥ 2		2.05	1.57–2.68	< 0.001	1.03	0.51–2.06	0.936	1.41	1.14–1.73	0.001	0.83	0.56–1.21	0.328
GNRI < 100		2.18	1.66–2.85	< 0.001	1.01	0.42–2.43	0.987	1.53	1.24–1.88	< 0.001	1.31	0.87–1.97	0.199
PNI < 45		2.23	1.47–3.36	< 0.001	1.19	0.55–2.57	0.651	1.32	0.97–1.79	0.075			
CONUT score ≥ 3		1.57	1.03–2.38	0.034	1.00	0.57–1.75	0.989	1.29	0.96–1.73	0.095			

HR, Hazard ratio; CI, Confidence interval; BMI, Body mass index; ICGR15, Indocyanine green retention rate at 15 min; DCP, Des-γ-carboxy prothrombin; ABC, Albumin **× **butyrylcholinesterase; ALBI, Albumin–bilirubin; GNRI, Geriatric nutritional risk index; PNI, Prognostic nutrition index; CONUT, Controlling nutritional risk status

In the univariate analysis, the significant prognostic factors for poor RFS included male sex, ICGR15 > 15%, AFP > 10 ng/mL, DCP > 400 mAU/mL, Child–Pugh B classification, multiple tumors, poorly differentiated or undifferentiated histology, extrahepatic metastasis, open liver resection, ABC < 951, ALBI grade ≥ 2, and GNRI < 100. In the multivariate analysis, the following were identified as prognostic factors for poor RFS: male sex (HR 1.36; 95% CI 1.04–1.78; *p* = 0.026), DCP > 400 mAU/mL (HR 1.53; 95% CI 1.18–1.99; *p* = 0.001), multiple tumors (HR 1.65; 95% CI 1.31–2.09; *p* < 0.001), poorly differentiated or undifferentiated histology (HR 1.52; 95% CI 1.07–2.16; *p* = 0.019), extrahepatic metastasis (HR 30.31; 95% CI 6.95–132.21; *p* < 0.001), and ABC < 951 (HR 1.44; 95% CI 1.04–1.99; *p* = 0.027).

### Early Recurrence in the Discovery and Validation Cohorts

**Fig. 3:**
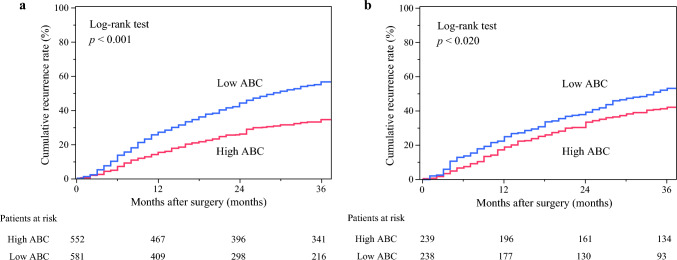
Cumulative early recurrence rate in the (**a**) discovery and (**b**) validation cohorts. ABC, albumin × butyrylcholinesterase

Figure 3 shows the recurrence rates within 3 years in both the discovery and validation cohorts. Significant differences in the recurrence rate between the low- and high-ABC groups were observed in both the discovery (*p* < 0.001; Fig. 3a) and validation (*p* = 0.020; Fig. 3b) cohorts.

### Survival Analysis and Early Recurrence for Patients with Liver Cirrhosis in the Discovery and Validation Cohorts

Figure S4 shows the Kaplan–Meier curves for OS, RFS, and the early recurrence rate, focusing on patients with liver cirrhosis in both cohorts. In the discovery cohort, OS, RFS, and the early recurrence rate differed significantly between the low- and high-ABC groups (Fig. S4a [*p* < 0.001], Fig. S4b [*p* < 0.001], and Fig. S4c [*p* = 0.018], respectively). However, in the validation cohort, significant differences in OS and RFS but not in the early recurrence rate were observed between the two groups (Fig. S4d [*p* = 0.003], Fig. S4e [*p* = 0.027], and Fig. S4f, [*p* = 0.762], respectively).

## Discussion

In this study, we designed a new marker to predict the prognosis for patients with HCC using Alb and BCHE levels. To our knowledge, this is the first study to show that the combination of serum Alb and BCHE is closely associated with the prognosis for patients with HCC.

To minimize time- and facility-related biases, we randomly divided the entire dataset obtained from seven facilities affiliated with the HiSCO in a 7:3 ratio. No significant differences in clinicopathologic characteristics were observed between the patients in the discovery and validation cohorts.

In the discovery cohort, the ABC cutoff value was determined using an ROC analysis of the OS. As a result, the Kaplan–Meier curves of the OS and RFS were stratified based on ABC in both the discovery and validation cohorts, identifying ABC as an independent risk factor for OS and RFS. Furthermore, the Kaplan–Meier curves of the cumulative early recurrence rate were stratified by ABC in both cohorts.

It is well-known that serum levels of Alb, a protein synthesized in the liver, reflect the nutritional status and liver function in patients. Serum Alb levels are used to calculate various nutritional and inflammatory indices, highlighting the importance of Alb as a prognostic indicator for patients with HCC. Also synthesized in the liver, BCHE is abundant in the serum, pancreas, liver, and central nervous system. Its serum levels decrease in various clinical conditions such as liver damage, inflammation, and malnutrition.^21^ Both Alb and BCHE may be influenced by preoperative treatments, particularly chemotherapy. It has been shown that cholinesterase (CHE) responds to chemotherapy in advanced gastric cancer.^22^ Findings have demonstrated low serum CHE levels to be a significant predictor of poor prognosis for patients with intermediate to advanced HCC undergoing treatment with sorafenib.^23,24^

In the entire cohort of this study, significantly more patients in the low-ABC group had received preoperative therapy (Table S1; *p* < 0.001). Subgroup analysis showed that regardless whether patients received preoperative therapy or not, the low-ABC group had a poor prognosis for both OS and RFS (Fig. S5). Furthermore, findings have shown that Alb and BCHE levels are associated with sarcopenia,^25,26^ which is significantly associated with impaired OS and a high risk of tumor recurrence for patients with HCC.^27^ These findings strongly suggest that this marker reflects patients’ overall condition and comorbidities. In the entire cohort of this study, a significantly higher number of patients in the low-ABC group had an American Society of Anesthesiologists Physical Status (ASA-PS) ≥ III (Table S2; *p* = 0.016).

This study did not examine the association between metabolic dysfunction-associated steatotic liver disease (MASLD) and metabolic dysfunction-associated steatohepatitis (MASH), which have been rising in prevalence recently and are known to increase the risk of overall and liver-related mortality. However, the significantly higher prevalence of liver cirrhosis in the low-ABC group suggests that this marker may reflect liver fibrosis, which is a component of the MASLD spectrum. Future research is required to investigate the association with MASLD and MASH.

We focused on patients with liver cirrhosis and performed the Kaplan–Meier analysis of OS, RFS, and the recurrence rate between the low- and high-ABC groups. As a result, significant differences in OS and RFS were observed in both cohorts, but no significant difference was found in the recurrence rate in the validation cohort. We believe this reflects the dual nature of ABC. In terms of tumor recurrence, the results of the subgroup analysis suggest that ABC strongly reflects liver function, whereas in terms of survival, it may also be strongly influenced by nutritional status.

It has been reported that liver dysfunction may be associated with the early recurrence of HCC. Hirokawa et al.^28^ showed that early recurrence after hepatectomy was associated with ICGR15 ≥ 16% and that risk factors of early recurrence vary by liver function status. Lee et al.^29^ showed that the ALBI score was a risk factor for early recurrence. As a result, it was suggested that liver dysfunction is associated with recurrence of HCC. Additionally, Alb and BCHE levels are related to tumor recurrence. Soreq et al.^30^ reported that phosphorylation by cdc2-related protein kinases, for which the target sites are present in both ACHE and BCHE, may be the molecular mechanism linking CHE with tumor cell proliferation. Thereafter, the associations between CHE and certain cancers were further investigated.^31,32^ Hirokawa et al.^33^ identified low CHE levels as a risk factor affecting RFS for patients with initial HCC who underwent hepatectomy. Additionally, adjuvant transcatheter arterial infusion therapy improved OS for patients with low CHE who had potential malignant factors. Amaoka et al.^34^ showed that vascular endothelial growth factor expression in non-tumor liver tissue was correlated with the values of serum Alb and CHE.

Regarding tumor markers, DCP levels in the discovery cohort were significantly lower in the low-ABC group. However, no significant difference was observed in the validation cohort (low ABC: 51 [23–332], high ABC: 43 [21–343]; *p* = 0.346). Therefore, ABC can be considered an independent risk factor, unrelated tumor markers.

In our study on prognostic indicators in HCC, we compared the ABC with general nutritional indices and liver function indicators in patients with HCC. We found that ABC was the most effective predictor of prognosis for patients with HCC. Furthermore, ABC is a simpler method that involves multiplying only two factors. In summary, ABC can be considered the simplest and most accurate prognostic indicator for HCC. Similar to its prognostic value for patients with colorectal cancer, ABC reflects the long-term prognosis for patients with HCC.^16^ However, there are several considerations when this marker is used. First, the cutoff value derived from the ROC curve in this study was 951. Although it is unlikely that values around the cutoff value would show dramatic differences in prognosis, in this study, the cutoff at 951 demonstrated the greatest prognostic impact.

Second, genetic mutations in BCHE have been reported to reduce baseline enzyme activity and potentially interact with certain drugs. The most common missense mutation, the K-variant (Ala567Thr, AS39T), has been associated with 30% lower BCHE activity compared with native BCHE.^35^ Although BCHE gene mutations are relatively rare, Lando et al.^36^ reported low plasma BCHE levels in 59 of 2609 healthy blood donors. This study<AQ4> was retrospective, and data on genetic factors related to BCHE activity or drug use were not collected. Therefore, the impact of these factors on prognostic prediction remains unevaluated. However, these factors are important, and future studies incorporating analyses that consider BCHE genetic diversity and drug use could potentially improve the accuracy of prognostic models.

Third, ABC values are considered reversible, indicating that improvements in Alb or BCHE levels could lead to better ABC values and potentially improve prognosis. Bi et al.^22^ demonstrated that CHE is associated with prognosis and response to chemotherapy in advanced gastric cancer, showing that patients whose CHE levels increased after chemotherapy had significantly higher survival rates than those with decreased levels. Further research and clinical evaluation are needed to determine the extent to which ABC improvement influences overall prognosis.

Our study had some limitations. First, it was a retrospective study and therefore was a subject to bias. Second, Alb and BCHE levels were evaluated only at the preoperative baseline. Third, all the data in this study were collected from institutions within a limited region. Further large-scale prospective studies are required to corroborate our findings.

In conclusion, ABC is an effective predictive biomarker for patients with HCC because it can be calculated easily and because it provides accurate prognostic information.

## Supplementary Information

Below is the link to the electronic supplementary material.

Supplementary file1 (TIF 89 KB)

Supplementary file2 (TIF 117 KB)

Supplementary file3 (TIF 120 KB)

Supplementary file4 (TIF 134 KB)

Supplementary file5 (TIF 116 KB)

Supplementary file6 (DOCX 16 KB)

Supplementary file7 (DOCX 15 KB)

## Data Availability

The data generated and analyzed in this study are available upon reasonable request.
