# Supplementary figures and images for: Albumin–Butyrylcholinesterase as a Novel Prognostic Biomarker for Hepatocellular Carcinoma Post-hepatectomy: A Retrospective Cohort Study with the Hiroshima Surgical Study Group of Clinical Oncology
Source: Ann Surg Oncol. 2024 Dec 10;32(3):1973–84. doi: 10.1245/s10434-024-16650-6 (PMC11811444; doi:10.1245/s10434-024-16650-6)

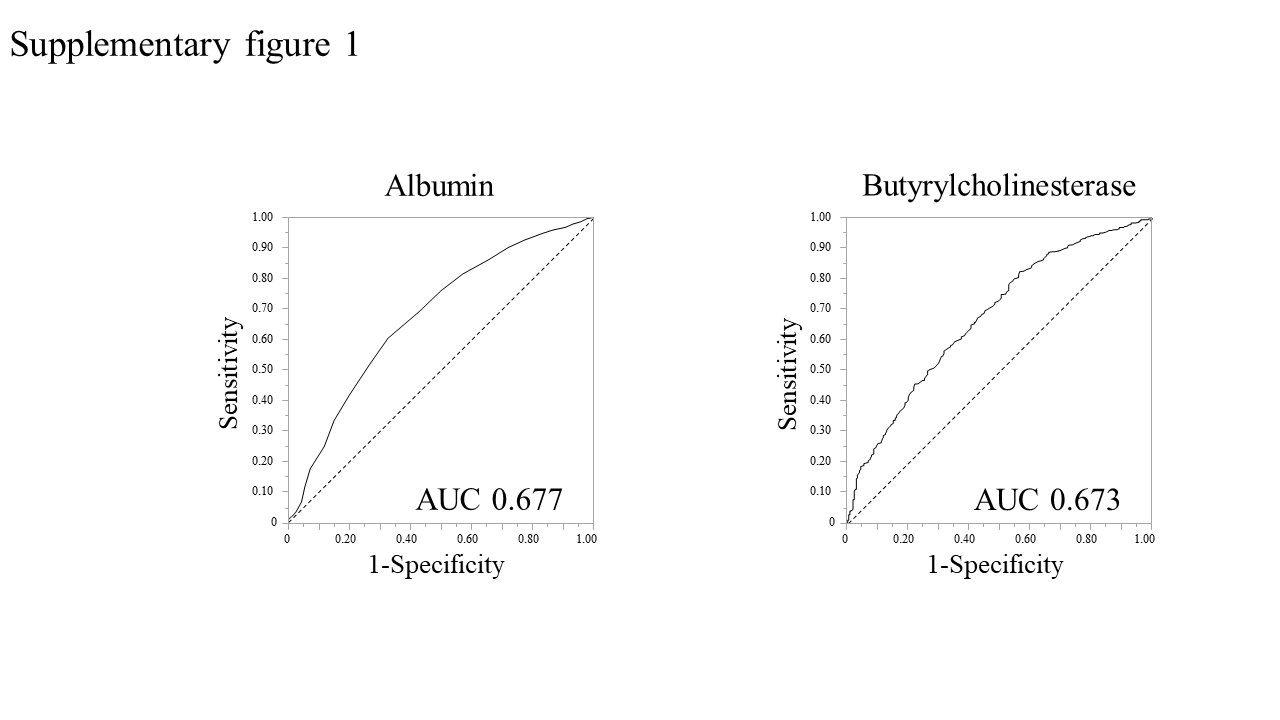

Supplement: Supplementary file 1 — Supplementary file1 (TIF 89 KB) [file 10434_2024_16650_MOESM1_ESM.tif]

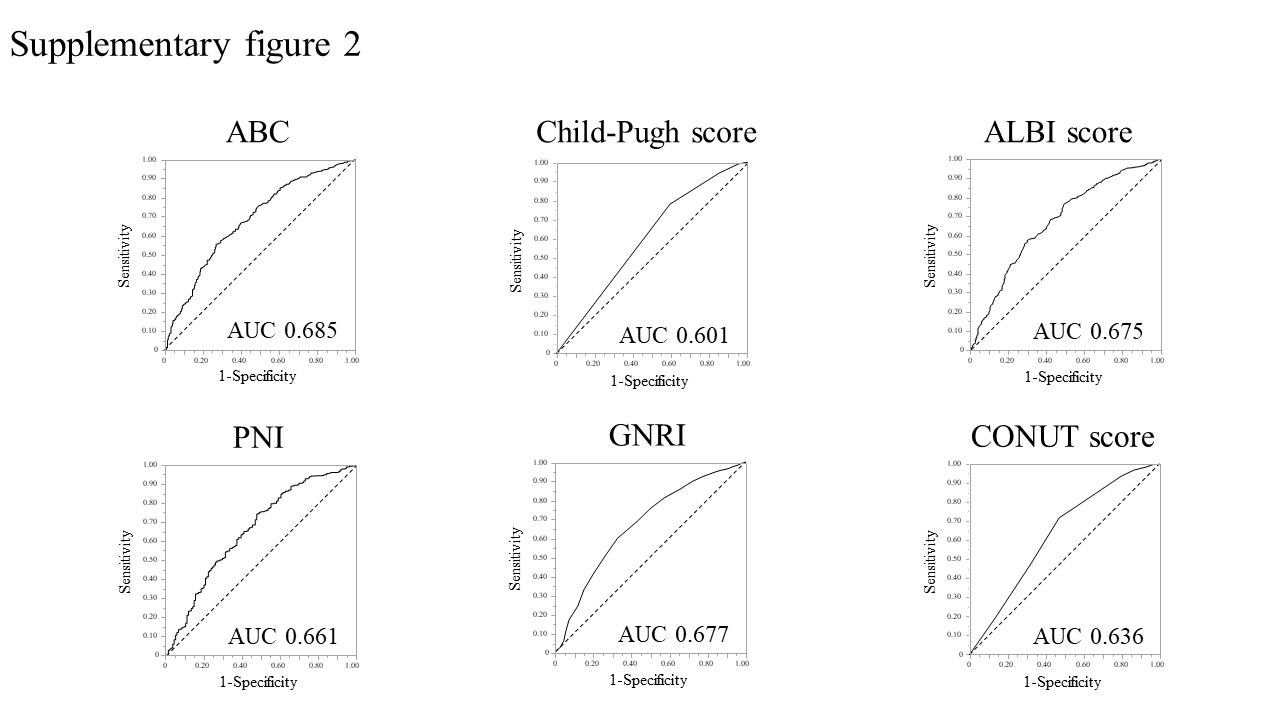

Supplement: Supplementary file 2 — Supplementary file2 (TIF 117 KB) [file 10434_2024_16650_MOESM2_ESM.tif]

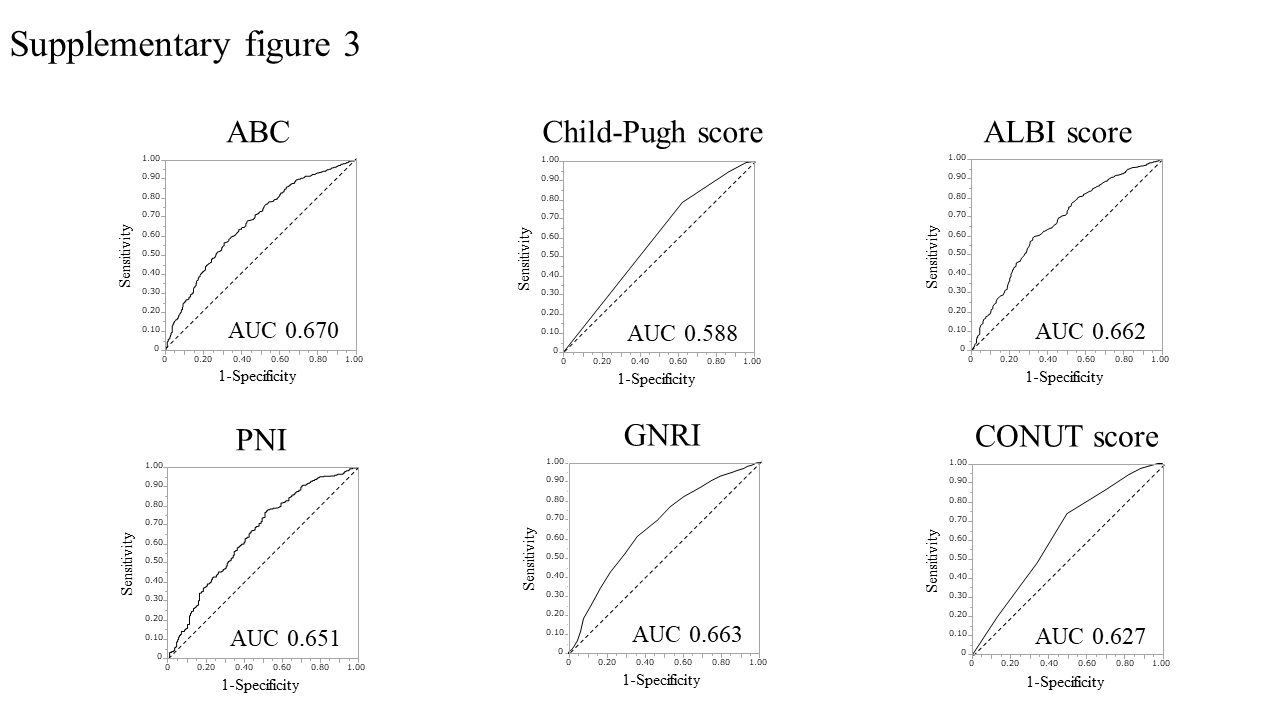

Supplement: Supplementary file 3 — Supplementary file3 (TIF 120 KB) [file 10434_2024_16650_MOESM3_ESM.tif]

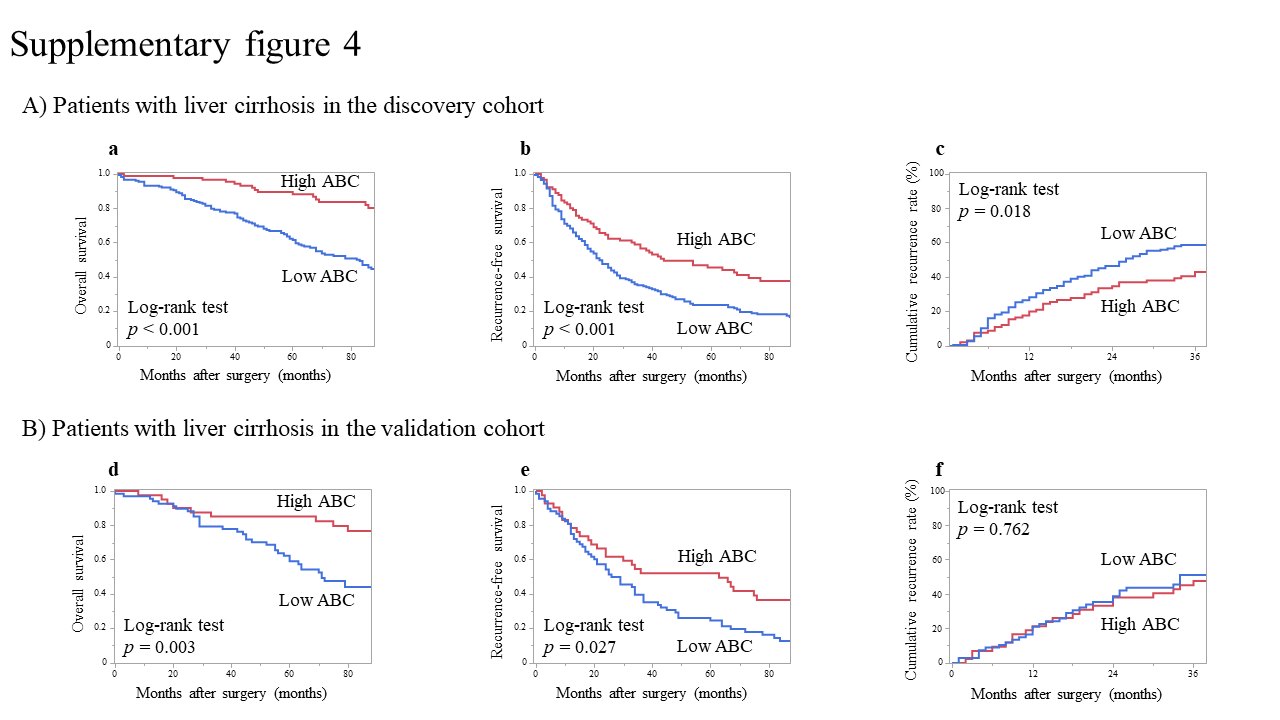

Supplement: Supplementary file 4 — Supplementary file4 (TIF 134 KB) [file 10434_2024_16650_MOESM4_ESM.tif]

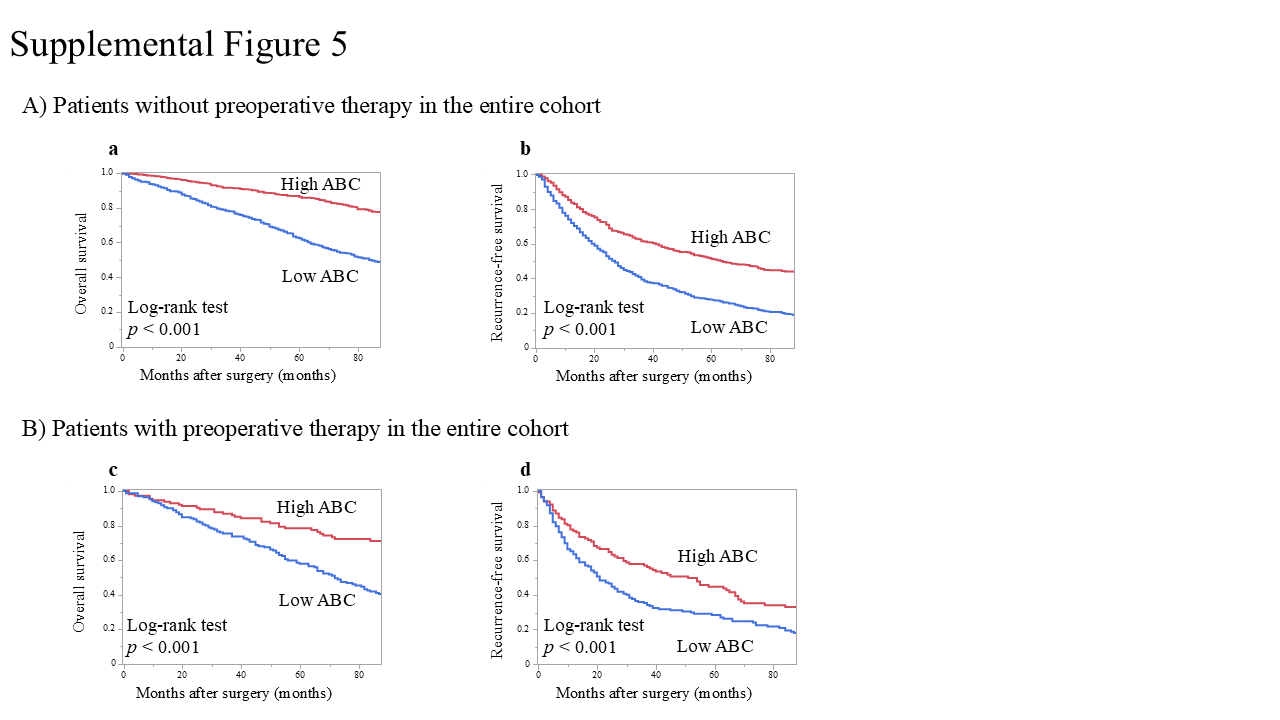

Supplement: Supplementary file 5 — Supplementary file5 (TIF 116 KB) [file 10434_2024_16650_MOESM5_ESM.tif]
