# Supplementary material for: Albumin–Butyrylcholinesterase as a Novel Prognostic Biomarker for Hepatocellular Carcinoma Post-hepatectomy: A Retrospective Cohort Study with the Hiroshima Surgical Study Group of Clinical Oncology
Source: Ann Surg Oncol. 2024 Dec 10;32(3):1973–84. doi: 10.1245/s10434-024-16650-6 (PMC11811444; doi:10.1245/s10434-024-16650-6)
Supplement: Supplementary file 6 — Supplementary file6 (DOCX 16 KB) [file 10434_2024_16650_MOESM6_ESM.docx]

| Supplemental Table 1. Comparison between low and high ABC groups in the entire cohort | | | | |
| --- | --- | --- | --- | --- |
| Variable |  | Low ABC group (n = 822) | High ABC group (n = 792) | p-value |
| Preoperative therapy | n (%) | 174 (21.2) | 117 (14.8) | < 0.001 |
| TAI | n (%) | 35 (7.3) | 17 (3.3) |  |
| TACE | n (%) | 53 (11.1) | 29 (5.7) |  |
| Unspecified TAI or TACE | n (%) | 86 (18.0) | 71 (14.0) |  |
| Variables are expressed as number (%) ABC, albumin**×**butyrylcholinesterase; TAI, Transcatheter arterial infusion; TACE, Transcatheter arterial chemoembolization | | | | |

| Supplemental Table 2. Comparison between low and high ABC groups in the entire cohort | | | | |
| --- | --- | --- | --- | --- |
| Variable |  | Low ABC group (n = 822) | High ABC group (n = 792) | p-value |
| ASA-PS ≥Ⅲ | n (%) | 62 (7.5) | 37 (4.7) | 0.016 |
| Variables are expressed as number (%) ABC, albumin**×**butyrylcholinesterase; ASA-PS, American Society of Anesthesiologists Physical Status | | | | |
