# Supplementary material for: Albumin–Butyrylcholinesterase as a Novel Prognostic Biomarker for Hepatocellular Carcinoma Post-hepatectomy: A Retrospective Cohort Study with the Hiroshima Surgical Study Group of Clinical Oncology
Source: Ann Surg Oncol. 2024 Dec 10;32(3):1973–84. doi: 10.1245/s10434-024-16650-6 (PMC11811444; doi:10.1245/s10434-024-16650-6)
Supplement: Supplementary file 7 — Supplementary file7 (DOCX 15 KB) [file 10434_2024_16650_MOESM7_ESM.docx]

**Supplemental material**

Supplemental Fig. 1: Receiver operating characteristics analysis of the overall survival in the discovery cohort.

Supplemental Fig. 2: Receiver operating characteristics analysis of the overall survival in the discovery cohort.

ABC, albumin × butyrylcholinesterase; ALBI, albumin-bilirubin; PNI, prognostic nutrition index; GNRI, geriatric nutritional risk index; CONUT, Controlling Nutritional Status; AUC, area under the curve.

Supplemental Fig. 3: Receiver operating characteristics analysis of the recurrence-free survival in the discovery cohort.

ABC, albumin × butyrylcholinesterase; ALBI, albumin-bilirubin; PNI, prognostic nutrition index; GNRI, geriatric nutritional risk index; CONUT, Controlling Nutritional Status; AUC, area under the curve.

Supplemental Fig. 4: Kaplan–Meier curves show (a) overall, (b) recurrence-free survival, (c) cumulative early recurrence rate in the discovery cohort, and (d) overall and (e) recurrence-free survival, (f) cumulative early recurrence rate in the validation cohort, in patients with liver cirrhosis.

ABC, albumin × butyrylcholinesterase

Supplemental Fig. 5: Kaplan–Meier curves show (a) overall, (b) recurrence-free survival without preoperative therapy, and (c) overall and (d) recurrence-free survival with preoperative therapy, in the entire cohort.

ABC, albumin × butyrylcholinesterase
